# Supplementary material for: Distribution and host range of viruses associated with the citrus leprosis disease complex in Mexico
Source: PeerJ. 2025 Sep 12;13:e19889. doi: 10.7717/peerj.19889 (PMC12435326; doi:10.7717/peerj.19889)
Supplement: Supplemental Information 1 — Primers were designed based on the sequence previously deposited in GenBank with the accession number NC_008170 [file peerj-13-19889-s001.doc]

**Table 1S.** List of primers used to sequence the RNA2 genomic segment of the virus CiLV-C found in lime leaves. Primers were designed based on the sequence previously deposited in GenBank with the accession number NC_008170.

| **Name** | **Sequence** | **Product length (bp)** |
| --- | --- | --- |
| R1-1F | TCA TCT ATT GTA TTG TTC TAG GCT | 958 |
| R1-1R | ATA TGT CAA CGC AAT CCG CTT C |  |
| R2-1F | GCG GAT TGC GTT GAC ATA TTC T | 1176 |
| R2-1R | ATC TGC GCA CTC AGT CCT AAA A |  |
| R3-1F | ATG GGC AGA AGC ATA TTG GTG AA | 934 |
| R3-1R | TCA GAC TGA ACG AGC ATT ACT |  |
| R4-1F | TCA CAG TAA TGC TCG TTC AGT | 840 |
| R4-1R | GTA TAC CAA GCC GCC TGT GA |  |
| R5-1F | TAA AGC TGA AGA CGC TGG GG | 1144 |
| R5-1R | ACG GCT GCC CCA ATG ATA AA |  |
